# The Effect of Aquaporin-4 Knockout on Interstitial Fluid Flow and the Structure of the Extracellular Space in the Deep Brain

**DOI:** 10.14336/AD.2017.1115

**Published:** 2018-10-01

**Authors:** Ze Teng, Aibo Wang, Peng Wang, Rui Wang, Wei Wang, Hongbin Han

**Affiliations:** ^1^Department of Radiology, Peking University Third Hospital, Beijing 100191, China; ^2^Beijing Key Lab. of Magnetic Resonance Imaging Technology, Beijing 100191, China; ^3^Department of Orthopedics, Peking University Third Hospital, Beijing 100191, China

**Keywords:** Aquaporin-4, interstitial fluid, extracellular space, tracer-based magnetic resonance imaging

## Abstract

It has been reported that aquaporin-4 (AQP4) deficiency impairs transportation between the cerebrospinal fluid and interstitial fluid (ISF) as well as the clearance of interstitial solutes in the superficial brain. However, the effect of AQP4 on ISF flow in the deep brain remains unclear. This study compared the brain ISF flow in the caudate nucleus and thalamus of normal rats (NO) and AQP4 knockout rats (KO) using tracer-based magnetic resonance imaging. The rate of brain ISF flow slowed to different degrees in the two regions of KO rats’ brains. Compared with NO rats, the half-life of ISF in the thalamus of KO rats was significantly prolonged, with a corresponding decrease in the clearance coefficient. The tortuosity of the brain extracellular space (ECS) was unchanged in the thalamus of KO rats. In the caudate nucleus of KO rats, the volume fraction of the ECS and the diffusion coefficient were increased, with significantly decreased tortuosity; no significant changes in brain ISF flow were demonstrated. Combined with a change in the expression of glial fibrillary acidic protein and AQP4 in two brain regions, we found that the effect of AQP4 knockout on ISF flow and ECS structure in these two regions differed. This difference may be related to the distribution of astrocytes and the extent of AQP4 decline. This study provides evidence for the involvement of AQP4 in ISF transportation in the deep brain and provides a basis for the establishment of a pharmacokinetic model of the brain’s interstitial pathway.

The extracellular space (ECS) of the brain is the narrow space between adjacent neural cells; its width ranges from 38 to 64 nm. The interstitial fluid (ISF) in the ECS provides survival environment for the nerve cells, delivers neurotransmitters and pharmaceutical agents, and removes metabolic wastes to or from the parenchyma [1]. The pattern of ISF flow through the ECS is controversial [2, 3]. ISF flow is a complex process related to many factors, such as age, brain activity, and structural characteristics within the ECS [1, 4, 5]. Related studies have indicated that ISF flow in the brain can also be affected by body posture, the extracellular matrix, and spontaneous hypertension [6-8]. The mechanism of ISF flow and ISF-cerebrospinal fluid (CSF) exchange in the superficial brain have been elucidated [9, 10]. Aquaporin-4 (AQP4) deficiency impairs the clearance of interstitial solutes in the ISF, and a decrease in its expression has been demonstrated in neurodegenerative diseases [2]. However, we still lack a comprehensive understanding of its function in physiological and pathological conditions. Given that the properties of ISF flow in the deep brain remain unclear, the mechanisms underlying encephalopathy have not yet been clarified in terms of the whole brain; thus, little progress has been made in terms of treatment.

The flow of ISF within the superficial cortex and its communication with the CSF have been widely explored and accepted [1, 2, 4, 9, 11]. However, at present, the functional mechanisms, hydromechanical characteristics, and components of ISF in the deep brain are not clear. Much research has focused on clarifying the properties and regulatory mechanisms of ISF flow in the deep brain [12-15], and it has been shown that external stimulation can affect ISF flow in the thalamus (Tha) [16]. AQP4, widely distributed on the foot processes of astrocytes, plays a critical role in interstitial flow [17]. Han et al. speculated that transportation in the deep brain is related to neuronal excitability and cell composition. During the neuronal excitation period, cell swelling of astrocytes in the Tha leads to volume reduction in the ECS and retards ISF flow [16]. Using a real-time iontophoresis method, Yao et al. found that the ECS volume fraction was increased in AQP4-deficient mice [18].

In the present study, we used tracer-based magnetic resonance (MR) imaging to compare differences in ISF flow between normal (NO) and AQP4 knockout (KO) rats in the caudate nucleus (Cn) and Tha, and used Western blotting to detect AQP4 expression. These data should allow us to elucidate the effect of AQP4 on ISF-CSF transportation in different brain regions, providing new insights for drug development and the treatment of brain diseases.

## MATERIALS AND METHODS

### Animals

This study was performed in accordance with the national guidelines for the use of experimental animals and the Helsinki Declaration, and the protocol was approved by the Ethics Committee of Peking University Health Science Center. Adult male Sprague Dawley rats and AQP4 KO rats (weighing 250-300 g) were used. Rats were singly housed under 12-h light/dark cycles. Temperature (22 ± 1 °C) and humidity (60 ± 5%) were controlled. Rats were anesthetized via an intraperitoneal injection of sodium pentobarbital (50 mg/kg), and anesthesia was maintained with ~30 mg/kg/h sodium pentobarbital during the experimental process.

### AQP4 KO Rat Model Establishment

The transcription activator-like effector nuclease (TALEN)-mediated KO approach was used to generate AQP4-deficient rats, as previously described [19, 20]. Briefly, we designed and synthesized highly active TALENs against the following sequences: (5’-CACA GCAGAGTTCCTGG-3’) for the sense strand and (5’-GGATCCCACGCTGAGCA-3’) for the antisense strand. The mRNAs of TALENs were injected into the cytoplasm of rat pronuclear stage embryos to produce mutant founders (F0). F0, which lacked three base pairs, were crossed with wild-type rats to produce the F1 generation. The heterozygous offspring of F1 were crossed to generate F2. Genomic analysis, conducted by sequencing polymerase chain reaction products, showed that the pups were heterozygous. Sanger DNA sequencing and Western blotting were used to confirm AQP4-deficiency, i.e., to confirm whether the TALEN-introduced AQP4 mutation was stably inherited. The AQP4 KO rats were viable and fertile and did not exhibit any gross abnormalities.

### Study Groups

The 12 AQP4-deficient rats in the KO group were randomly divided into Cn group (*n* = 6) and Tha group (*n* = 6). The 12 normal rats in the NO group were also randomly divided into these two subgroups.

### MR Imaging Scan Protocols

A 3.0 T MR imaging system (Magnetom Trio, Siemens Medical Solutions, Erlangen, Germany) with an eight-channel wrist coil was used to obtain rat brain images by running a T1-weighted magnetization-prepared rapid-acquisition with gradient echo (MP-RAGE) sequence. The acquisition parameters were as follows: echo time = 3.7 ms, repetition time = 1500 ms, flip angle = 12°, inversion time = 900 ms, field of view = 267 mm, voxel = 0.5 mm^3^, matrix = 512 × 512, number of averages = 2, phase-encoding steps = 96. The acquisition time for each rat was 290 seconds. For each subject, scanning was performed before and after the introduction of gadolinium-diethylenetriaminepentaacetic acid (Gd-DTPA). The scan time points were set as pre-injection and at each time point post-injection until the “bright region” faded.

### Stereotaxic Intracranial Injections of Tracer in Tracer-Based MR Imaging

Gd-DTPA (Magnevist; Bayer Schering Pharma AG, Berlin, Germany)—which cannot be taken up by neural or neuroglial cells—was diluted to 10 mmol/L with 154 mmol/L sodium chloride solution; 2 μL of this diluted Gd-DTPA was then injected into assigned positions of the brain. After injection, the signal intensity decreased rapidly.

MR imaging was conducted before administering Gd-DTPA so as to determine the injection route and depth. Each rat was anesthetized, and rectal temperatures were monitored with a rectal thermometer; their temperatures were maintained at 38 ± 0.5°C using heating pads. Additionally, other vital signs (blood pressure, heart rate, and respiratory rate) were also monitored; no significant differences were observed between the groups. The skin covering the calvaria was shaved and disinfected with iodophor solution. An incision was made in the scalp along the sagittal suture from the interaural area to the interocular area. The membranes and muscle attachments were dissected free of the skull bone, and the bregma suture was exposed. The rat was immobilized in a stereotactic coordinate system (Lab Standard Stereotaxic-Single, Stoelting Co, Illinois, USA) and a small trephine hole was made according to the stereotactic coordinates of Tha (bregma: -3.0 mm, lateral: 2.0 mm) or Cn (bregma: +1.0 mm, lateral: 3.5 mm). Microsyringe needles were inserted to a depth of 6.0 mm and 5.0 mm, respectively. Gd-DTPA was employed as an extracellular probe to trace the dynamic distribution of brain ISF. A total volume of 2 μL of the tracer (10 mmol/L) was delivered into the ECS of the brain via a 10 μL microsyringe (Hamilton, Bonaduz AG, Switzerland) at a rate of 0.2 μL/min using an automated drug administration system (Harvard Apparatus, USA). The infusion was followed by a 5-min waiting period in case dorsal reflux occurred along the needle track. The rat was then quickly placed in the scanner in a prone position for the post-injection scan, performed according to the MR imaging scan protocols.

### Post-Procedure Image Processing

MATLAB-based software was developed to co-register the MR images of each rat pre- and post-injection. Post-injection images were compared with the baseline images after gray scale calibration, image registration based on mutual information, and histogram equalization. All images obtained post-injection were automatically subjected to rigid transformation, similarity measurements, high-order interpolation, and adaptive stochastic gradient descent optimization. These images were then subtracted from the pre-scanned images. The acquired “bright areas”, obtained by establishing a seed point and a threshold in the regions of interest, were assumed to be related to the presence of the tracer. New sets of post-processing MR images in the horizontal, sagittal, and coronal planes with slice thicknesses of 1 mm were generated by the software. After the co-registration and subtraction process, the signal intensity within the target area of the processed images was measured and denoted by ∆SI; this was used to calculate the diffusion parameter in the ECS of the rats’ brains.

### Calculation of the ISF diffusion parameters and the ECS structure parameters

The brain tissue around the injection site appeared as a high-intensity spot on the MR image shortly after injection of the tracer solution. Enhancement of the MR signal intensity caused by the tracer was converted to its concentration using an empirical fitting process. Thus, both the diffusion parameters of the ISF and structural parameters of the ECS could be calculated based on the obtained distribution of the tracer concentration. According to the modified diffusion equation, the equivalent diffusion coefficient (D*) and clearance parameters (k’) in each MR image pixel near the injection site (1-2 mm from the site) could be derived. Because the clearance of the tracer in the whole rat brain fitted well with a mono-exponential decay function, the k’ and half-life (t½) could be used to represent both the clearance rate and transportation speed. The volume fraction (α) is defined as the volume fraction of ECS in the whole brain tissue. Tortuosity is defined as λ = $\sqrt{D/D*}$, where D* is the effective diffusion coefficient of a given molecule in the brain ECS, D is the diffusion coefficient of the same molecule in a free medium, λ is the hindrance to diffusion imposed by local structure of the ECS, and Q is the source that is released into the ECS [21].

### Western Blotting

Glial fibrillary acidic protein (GFAP) and AQP4 levels in the Cn and Tha of rat brains were detected by Western blotting, as previously described [22]. Briefly, protein lysates were obtained using RIPA lysis buffer (Beyotime, Shanghai, China) and 50 μg of each sample was loaded onto a 12% tricine-sodium dodecyl sulfate-polyacrylamide gel and subsequently transferred to a polyvinylidene difluoride membrane (Bio-Rad, Hercules, CA, USA). Membranes were blocked for 1 h with 5% nonfat milk and incubated overnight at 4°C with primary antibodies against GFAP (rabbit anti-rat; 1:1000; Abcam) and AQP4 (rabbit anti-rat; 1:1000; Abcam) or a monoclonal antibody against β-actin (1:10,000; Abcam) in TBST (10 mmol/L Tris, pH 8.0, 150 mmol/L NaCl, 0.05 % Tween 20) containing 3% nonfat milk. Membranes were then incubated for 1 h at room temperature with secondary antibody (goat anti-rabbit; 1:1000; Abcam). Proteins were detected by chemiluminescence and analyzed using Image J software (National Institutes of Health, Bethesda, MD, USA).

**Figure 1.**
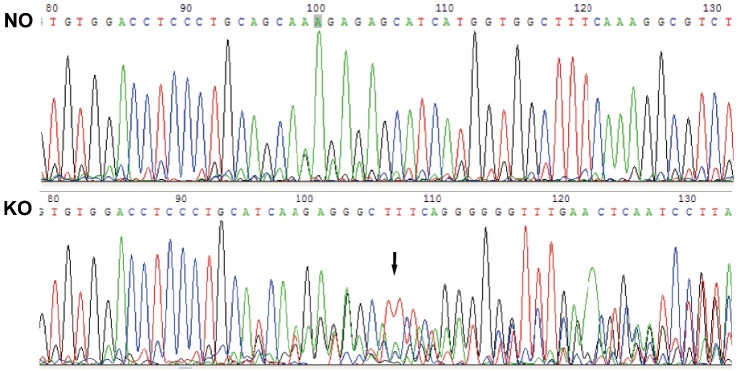
Peak map of AQP4 gene sequencing of normal (NO) and AQP4 knockout (KO) rats Compared with NO rats, AQP4 KO rats showed a multimodal peak near the AQP4-T1 position (black arrow), indicating AQP4 gene deletion.

### Statistical Analysis

Statistical analyses were performed using IBM SPSS software, version 19.0 (IBM Corp, Armonk, NY). The data were expressed as the mean ± standard deviation (SD). Independent-sample *t*-tests were used to compare the diffusion parameters of ISF, structural parameters of the ECS, and the expression of GFAP and AQP4 in the NO group and KO group. A p-value < 0.05 was considered statistically significant.

## RESULTS

### Verification of the AQP4 KO Rat Model

AQP4 KO rats were established using the TALEN technique, and successful germline inheritance resulted in stable F1 gene KO rats. The TALEN technique is used to edit DNA (including performing knock out, knock in, base substitution, mutations, or gene modifications) after fusion protein, combination of TALEs (transcription activator-like effectors) with the nuclease Fok I, splits the target gene DNA sequence at specific sites. Comparison of gene sequencing peaked with NO rats; AQP4 KO rats showed a multimodal peak near the AQP4-T1 position, indicating a frameshift mutation in the AQP4 gene and inactivation of the AQP4 protein, resulting in the deletion of the AQP4 gene (Fig. 1).

### Effect of AQP4 on ISF Flow and ECS Structure

Gd-DTPA probes were injected into the Cn and Tha respectively. Due to the longitudinal relaxation effect of the probe, the free water molecules in the ISF are labeled and display as high signals on the MR image. When the tracer spreads to the ECS and drains with the ISF, the signal intensity gradually decrease, and the distribution area increases. MR imaging showed that the t½ in the Tha of KO group was prolonged in comparison with that of the NO group (82.83 ± 6.95 vs 52.60 ± 6.87 min, *p*<0.05) and the tracer’s direction of flow differed in the Cn and Tha regardless of KO. However, the t½ was not significantly different in the Cn of KO group and NO group (96.69 ± 11.05 vs 86.93 ± 12.28 min, *p*>0.05). The ISF in the Cn flowed to the cortex of the ipsilateral frontal and parietal lobes, and finally into the subarachnoid space. In the Tha, however, ISF flow was limited to that anatomical region—it did not diffuse or flow to other brain regions. Although the Cn is in close anatomical proximity of the Tha, the ISF in the Cn did not flow to the Tha, and vice versa.

**Figure 2.**
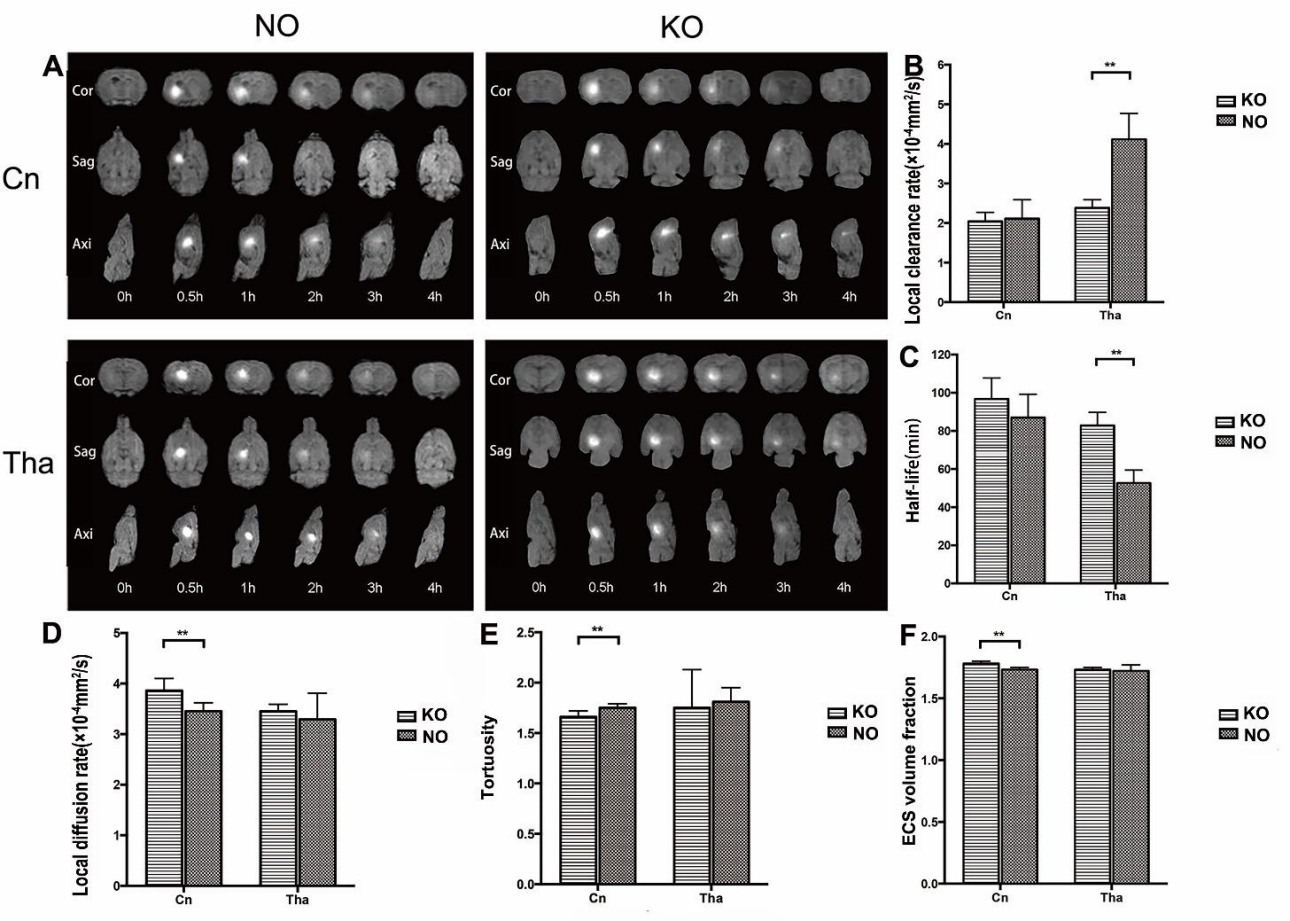
Flow of interstitial fluid and the structure of the extracellular space in the caudate nucleus and thalamus of normal (NO) and AQP4 knockout (KO) rats **(A)** Interstitial fluid flow was visualized by tracer-based magnetic resonance imaging and is shown in sagittal, axial, and coronal views. The interstitial fluid in the caudate nucleus flowed to the prefrontal and parietal cortex and finally poured into the cerebrospinal fluid. Interstitial fluid in the thalamus was restricted within its anatomical region. **(B-F)** In the caudate nucleus, diffusion parameters and the volume fraction of the ECS were increased in the KO group and tortuosity was decreased, but no differences were found in the local clearance rates and half-life. In the thalamus, the local clearance rates in the KO group were less than those of the NO group, the half-life in the KO group was prolonged. No difference was found in the diffusion parameters, volume fraction, and tortuosity of ECS. The data are expressed as the mean±standard deviation (SD). n=6 rats per group, **P<0.05.

The ultrastructure of the ECS and the micro-distribution process of ISF were calculated using post-processing computer software. Compared with the NO group, the α of ECS in the Cn of the AQP4 KO group was increased (1.78 ± 0.02 vs 1.73 ± 0.02, *p*<0.05), with significantly decreased λ (1.66 ± 0.06 vs 1.75 ± 0.04, *p*<0.05) and a significantly increased D* (3.86 ± 0.24 vs 3.45 ± 0.17 × 10^-4^ mm^2^/s, *p*<0.05) values. In the Tha of rats in the AQP4 KO group, the α (1.73 ± 0.02 vs 1.72 ± 0.05, *p*>0.05), λ (1.75 ± 0.38 vs 1.81 ± 0.14, *p*>0.05) and D* (3.45 ± 0.14 vs 3.29 ± 0.52 × 10^-4^ mm^2^/s, *p*>0.05) values did not change significantly, but the k’ of the ISF tracer decreased significantly (2.38 ± 0.21 vs 4.11 ± 0.66 × 10^-4^ mm^2^/s, *p*<0.05). Compared with the NO group, the k’ in the Cn of the AQP4 KO group was not significantly different (2.04 ± 0.23 vs 2.11 ± 0.48 × 10^-4^ mm^2^/s, *p*>0.05) (Fig. 2).

### Expression of GFAP and AQP4 in the Cn and Tha

**Figure 3.**
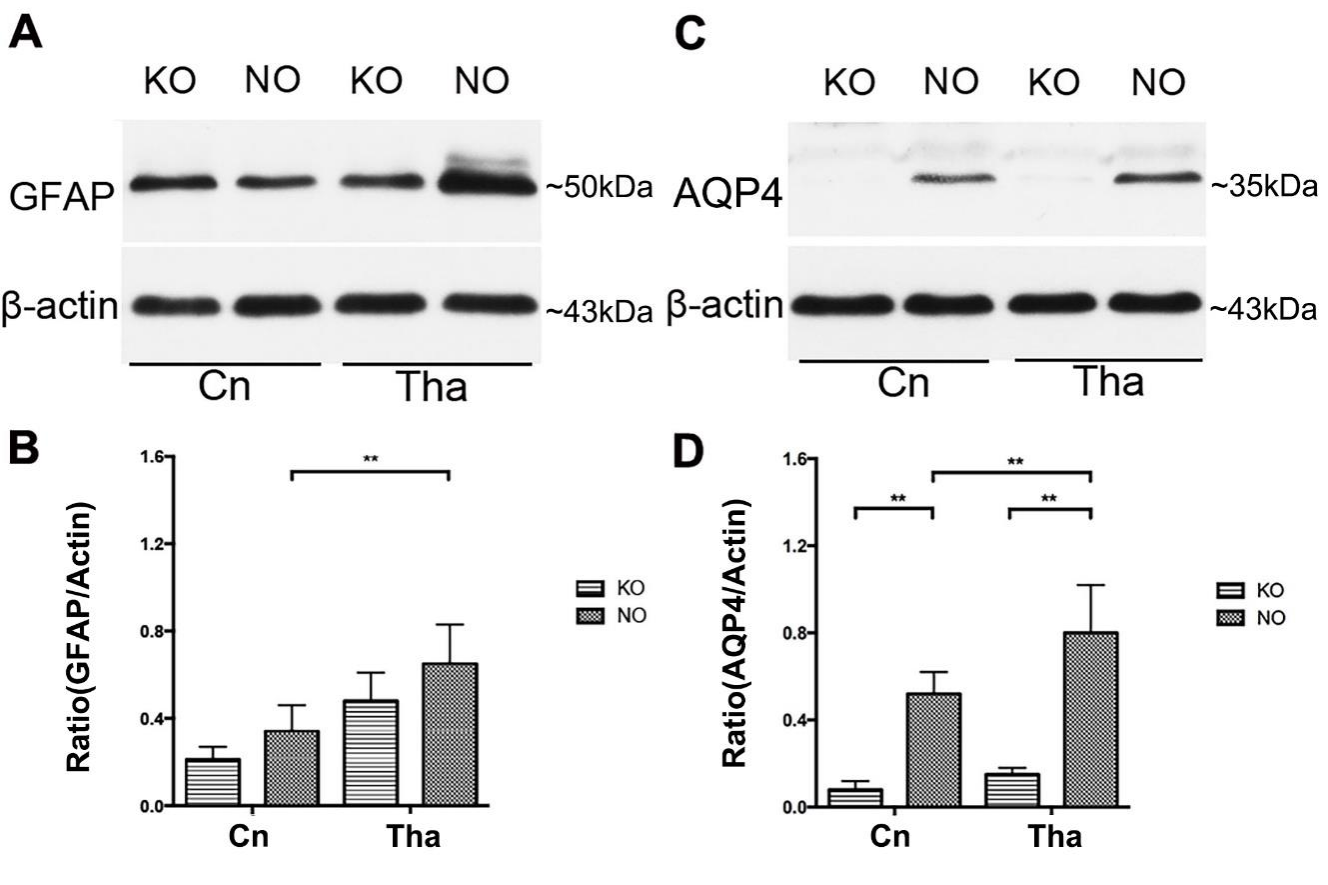
Protein expression of GFAP and AQP4 in the caudate nucleus and thalamus **(A, C)** The expression of glial fibrillary acidic protein (GFAP) and aquaporin-4 (AQP4) in the thalamus was significantly higher than that in the caudate nucleus in the normal (NO) group. **(B, D)** AQP4 was weakly expressed in the caudate nucleus and thalamus in the AQP4 knockout (KO) group, whereas GFAP expression did not change significantly in either region. The data are expressed as the mean ± standard deviation (SD). n=6 rats per group, **P<0.05.

The levels of GFAP and AQP4 expression in the Cn and Tha of the NO and KO rat groups were detected by Western blotting. In the NO group, the expression of GFAP (0.65 ± 0.18 vs 0.34 ± 0.12, *p* <0.05) and AQP4 (0.80 ± 0.22 vs 0.52 ± 0.10, *p* <0.05) was significantly higher in the Tha than in the Cn. In the AQP4 KO group, AQP4 was weakly expressed in the Cn (0.08 ± 0.04 vs 0.52 ± 0.10, *p* <0.05) and Tha (0.15 ± 0.03 vs 0.80 ± 0.22, *p* <0.05). GFAP expression did not change significantly in either region (Cn: 0.21 ± 0.06 vs 0.34 ± 0.12, *p*>0.05; Tha: 0.48 ± 0.13 vs 0.65 ± 0.18, *p* >0.05) (Fig. 3).

## DISCUSSION

AQP4, the richest bidirectional aquaporin expressed in the brain, is involved in water transport, cell migration, and neuronal excitability [23]. It is of great significance for the balance of water within the intercellular space and the realization of brain function [24]. AQP4 primarily localizes to the subpial astrocyte processes, the perivascular astrocyte endfeet, the basolateral membrane of ependymal cells, and subependymal astrocyte processes [2, 23, 25-27]. Normally, water molecules enter the cell facilitated by AQP4, causing astrocyte swelling and ECS volume reduction [23]. AQP4 KO mice have normal glial cell characteristics; however, their astrocyte volume regulation capacity is impaired [28] and cell membrane water permeability is decreased; this leads to an increase in ECS water volume [29] and a mildly raised ECS volume ratio [18].

**Figure 4.**
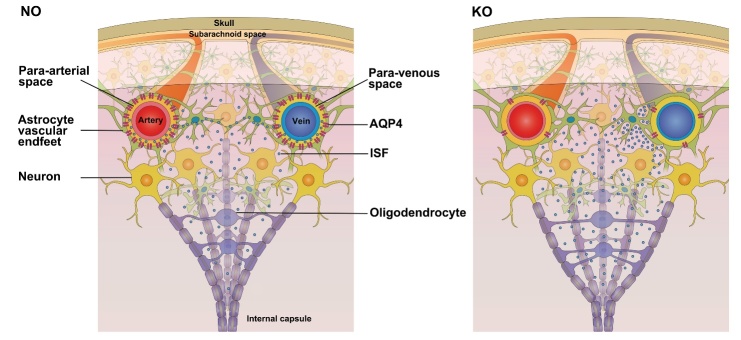
Changes of in ISF flow in the ECS between the caudate nucleus and cortex In normal deep brain, ISF flows from the caudate nucleus to the cortex along myelinated fibers, whereas it is cleared from the brain along paravenous routes in the cortex. The flow of ISF is facilitated by AQP4-dependent astroglial water flux, which drives fluid dispersion into the subarachnoid CSF. In the brains of knockout rats, AQP4 deficiency led to obstruction of ISF clearance and accumulation of ISF in the cortex and caudate nucleus, resulting in a decrease in tortuosity and an increase in the volume fraction and molecular diffusion rate in the ECS within the caudate nucleus.

This study compared the flow of ISF in the deep brain of NO and KO rats. AQP4 deficiency influenced ISF flow and ECS structure, which differed from findings in the cortex in previous studies [2]. After gene knockout, the decreasing extents of ISF flow characteristics in two regions of the brain differed, which might be because of distinct flow pathways. Using a dynamic, tracer-based MR imaging method, we found that the ISF flow pathways in the Cn and Tha were different in NO rats. In the Cn, ISF flowed to the prefrontal and parietal cortex and finally poured into the CSF. However, in Tha the ISF clearance was restricted to that anatomical region. Compared with Tha, the ISF in the Cn flows along a relatively long pathway and would be more susceptible and sensitive to flow changes in downstream regions. Moreover, previous studies indicated that, in KO rats, the exchange of ISF-CSF and the efflux efficiency of ISF were reduced in the cortex [2]. Thus, the ISF flow from the Cn to the cortex might be obstructed, resulting in accumulation of ISF in the deep brain. This accumulation manifested as a decline in ISF flow velocity, but this decline was not statistically significant. Nonetheless, this slight decline led to an increase in the volume fraction [29], a decrease in tortuosity, and an increase in the molecular diffusion rate in the ECS of the Cn (Fig. 4). This suggests that the variation in ECS and ISF flow once AQP4 was lacking differed between the Tha and Cn.

Compared with the NO rats, the ISF flowed significantly slower and the decline in the microscopic clearance rate differed significantly in the Tha of AQP4 KO rats. These results suggest that the decline in ISF flow ability may be related to the decline in clearance rate. The difference between the Tha and Cn might be due to the discrepancy in the proportion of various cells in different brain regions. We found that in NO and KO rats, the expression of GFAP in Tha was significantly higher than that in Cn. This confirms that the proportion of astrocytes in Tha is greater than that in Cn, which is important given that AQP4 is distributed mainly on the endfeet of astrocytes [30, 31]. Thus, in KO rats, the extent of AQP4 expression decline is much more obvious in the Tha than in the Cn, which could partially explain the phenomenon that in the Tha ISF clearance declined much more significantly. After AQP4 knockout, the volume fraction and molecular diffusion of ECS were only slightly increased in the Tha. Although these increases were not statistically significant, the impact on ISF flow was most significant. This suggests that the ECS microstructure is not the principal factor affecting ISF flow, rather, it is a composite of the incremental volume fraction increase and the cell membrane permeability decrease that effects changes in microscopic diffusion in KO rats [32]. Thus, the variations in the microstructure and cell characteristics in different brain regions might result in the observed discrepancy in the microscopic diffusion rates between the Cn and Tha.

Our results suggest that the microscopic clearance rate is the main factor affecting ISF flow in the nuclei of the deep brain and that the effects of AQP4 KO on the structure of the ECS and on ISF flow differ according to brain region. Combined with previous research findings, this study further elucidates how AQP4 affects brain ECS structure and ISF flow, and highlights the biological and pathological functions of AQP4 in the brain. Our data could contribute to simulating the transport process of polar molecular drugs, providing parameters for the pharmacokinetic model of drug distribution in the future, and prompting new thoughts and possible methods for regulating drug distribution in the brain.
